# Perceptions on healthy aging: insights from focus group interviews of older adults in Sweden

**DOI:** 10.1186/s13690-026-01915-1

**Published:** 2026-04-16

**Authors:** Nathalie Frisendahl, Ann Liljas, Mariam Kirvalidze, Janne Agerholm, Anna-Karin Welmer, Amaia Calderón-Larrañaga

**Affiliations:** 1 Division of Physiotherapy, Department of Neurobiology, Care Sciences and Society, Karolinska Institutet, Stockholm, Sweden; 2 Department of Global Public Health, Karolinska Institutet, Stockholm, Sweden; 3 Aging Research Center, Department of Neurobiology, Care Sciences and Society, Karolinska Institutet and Stockholm University, Stockholm, Sweden; 4 Stockholm Gerontology Research Center, Stockholm, Sweden

**Keywords:** WHO Healthy Ageing Framework, Social support, Continuous care, Autonomy, Social and physical environment

## Abstract

**Background:**

As populations age rapidly, understanding the factors influencing healthy aging is essential. This study explores older adults’ perceptions of healthy aging to broaden perspectives and contextualize the concept within the World Health Organization (WHO) Healthy Ageing Framework.

**Methods:**

Thirty-four participants aged ≥ 60 years from the population-based Swedish National study on Aging and Care in Kungsholmen (SNAC-K) were interviewed through seven semi-structured qualitative focus groups between December 2023–January 2024. Discussions focused on participants’ perspectives on healthy aging, including factors that facilitate or hinder their ability to age as they desire. Data were analyzed using reflexive thematic analysis.

**Results:**

Two overarching themes were identified: “Inner power through vitality and motivation” and “A sense of being needed and having one’s needs met.” Participants emphasized the importance of maintaining autonomy, adapting to life transitions, fostering social connections, and navigating societal structures that impact healthy aging.

**Conclusions:**

The findings largely align with the WHO Healthy Ageing Framework, reinforcing the significance of social support, continuous care, and autonomy. By highlighting the importance of an individual’s social and physical environment on their health, this study contributes to an often-overlooked perspective acknowledged by the WHO Healthy Ageing Framework. Participants’ concerns about the current and long-term capacity of the welfare system underscore the urgent need for targeted policy interventions to support the rapidly growing older population. Additionally, the findings emphasize the need to promote equal opportunities among older adults, as those in good health can leverage their inner motivation to build and lead meaningful lives.

**Supplementary Information:**

The online version contains supplementary material available at 10.1186/s13690-026-01915-1.

**Table:** 

Text box 1. Contributions to the literature
• Highlights how older adults’ experiences of healthy aging can inform population-level health strategies.
• Emphasizes the importance of autonomy, motivation, and supportive environments for maintaining health in later life.
• Draws attention to structural and policy factors affecting older adults’ well-being, supporting targeted interventions.
• Offers practical insights for aligning public health policies with older adults’ lived experiences and priorities.

## Introduction

Worldwide, and also in Sweden, populations are aging due to increasing life expectancy and low birth rates [1]. By 2030, nearly 25% of the population will be aged 65 years or older, with the most significant growth occurring among individuals aged 80 years and above [2]. This demographic shift places considerable pressure and increased costs on the healthcare system, as Sweden’s health expenditure is among the highest in the OECD, accounting for approximately 11.3% of GDP and around USD 7,800 per capita, both above OECD averages [3]. In a publicly funded welfare system such as Sweden’s, these demographic changes raise concerns about the long-term capacity of health and social care services to meet growing needs. As the oldest individuals typically require the most care, this development may contribute to unmet healthcare needs [4].

In Sweden, health and social care for older adults is primarily publicly funded and organized through a decentralized welfare system. Healthcare services are mainly provided by regional authorities, while municipalities are responsible for social care, including home care services and residential care facilities. A central policy principle is to support older adults in remaining in their own homes for as long as possible through home care services, with admission to nursing homes being needs-based and typically reserved for individuals with extensive care needs. As a result, access to nursing home care is limited, and shortages of available places have been reported [5].

Within this policy context, where responsibility for healthy aging is shared across health and social care services, and the broader living environment, understanding how healthy aging is conceptualized and operationalized becomes particularly important [6]. The World Health Organization (WHO) Healthy Ageing Framework offers a useful lens for examining how individual capacities interact with health, social and environmental conditions within welfare-state contexts such as Sweden’s. In this Framework, healthy aging is defined as “the process of developing and maintaining the functional ability that enables well-being in older age” [7]. This comprehensive definition implies accounting for both internal biological and biographical factors such as genetics, gender, and ethnicity, as well as external social and physical environmental influences. Previous research shows that social and cultural factors also shape how people understand and adopt the concept of healthy aging [8]. Hence, functional ability is influenced by a combination of physical health, knowledge, skills, and both social and environmental factors. By addressing these determinants, the Framework seeks to create environments that empower individuals to lead fulfilling lives while maintaining their independence as they age [9]. The Framework also underscores the importance of cross-sector collaboration**—**healthcare services, education, and urban planning**—**to develop sustainable solutions that support healthy aging. Despite its growing use in quantitative research, the WHO Healthy Ageing Framework has been explicitly applied in relatively few qualitative studies, particularly in community settings [10].

Understanding the health needs and experiences of older adults provides critical insights into their priorities [11]. To ensure that aging research addresses what matters most to them, it is essential to integrate their perspectives into research, clinical guidelines, and health resources**—**a practice that has been inconsistently applied [12, 13]. These perspectives are crucial for developing effective preventative strategies and advancing person-centered care [14, 15]. This study aims to explore how older adults perceive healthy aging. Although an open analytical approach was employed, free from predetermined assumptions, the findings are subsequently situated and discussed in relation to the WHO Healthy Ageing Framework.

## Methods

This is an exploratory study with a qualitative approach for which we collected focus group interview data [16] in Stockholm, Sweden. The Reflexive Thematic Analysis Reporting Guidelines (RTARG) [17, 18] were followed to guide the study, as well as The Standards for Reporting Qualitative Research (SRQR) checklist (Additional file 1).

### Participants

Participants in this study were drawn from the ongoing longitudinal, population-based Swedish National study on Aging and Care in Kungsholmen (SNAC-K, snac-k.se), which includes individuals aged 60 years and older [19]. Initiated in 2001, SNAC-K employed a stratified sampling method. The population of Kungsholmen, a central relatively affluent district of Stockholm, was initially stratified by age, followed by the selection of a random sample from each age cohort: 60, 66, 72, 78, 81, 84, 87, 90, 93, 96, and 99 years and older.

Between 2001 and 2004, 3,363 individuals participated in the baseline examination, representing 73.3% of those eligible [19]. Participants are reassessed at six-year intervals in the younger age groups (60–72 years) and at three-year intervals for those aged 78 years and older. New cohorts of individuals aged 60 and 81 years have been added since 2007–2010 (wave 3). Assessments include clinical examinations, cognitive evaluations, and interviews conducted by physicians, nurses, and psychologists.

### Procedure

Recruitment to the current study took place in a lecture hall during the SNAC-K proband day at Karolinska Institutet, Sweden, in October 2023. This event was designed to share research findings with participants. SNAC-K probands interested in participating in our study were encouraged to register their name and telephone number at a desk outside the lecture hall on that day. Through this self-referral convenience sampling [16], a total of 60 SNAC-K participants expressed their interest and provided information about their age, gender, highest level of education (elementary/high school/university) and the year they enrolled in the SNAC-K study (Table 1).

**Table 1:** List of focus group participants and their demographic information

Focus group	Age range (years)	Female	Male	University	High school	Elementary school	Unknown educational level
1 (*n* = 6)	63–73	5	1	5	1	0	0
2 (*n* = 6)	63–73	5	1	4	2	0	0
3 (*n* = 5)	81–88	3	2	4	1	0	0
4 (*n* = 5)	71–73	4	1	5	0	0	0
5 (*n* = 4)	80–86	0	4	3	0	0	1
6 (*n* = 3)	81–88	3	0	2	0	0	1
7 (*n* = 5)	72–82	5	0	0	4	1	0

Based on this information, a total of seven focus groups (total *n* = 34 participants) were formed. In focus groups, participants typically share certain characteristics [16]. In the first round of focus groups, the participant information provided was used to categorize groups by age (one group with participants aged > 75 years and three groups aged 60–75 years), balanced by gender (at least one male participant per group) and a mix of educational levels across all groups. In the following weeks, researcher NF contacted participants by telephone to explain the study, answering any questions, and asked about their interest in participating in a focus group on a specific day and time at the Aging Research Center of Karolinska Institutet. Selection was guided not only by age, gender and education but also by availability on the day of the focus group. Non-responders received follow-up reminders after 4–6 weeks, and all participants allocated to a focus group interview (referred to as FG after each verbatim) received a text message reminder one week prior.

We aimed to generate a dataset rich in perspectives across age, gender, and educational background to support a nuanced, interpretative analysis [20]. Therefore, in a second round of recruitment (based on the enrolment list), we purposively selected individuals aged over 75 years (two groups), and men (one group). The same recruitment procedure applied for the second round of focus group participants. The size and composition of the participant groups were found to be sufficient to develop detailed and meaningful themes in line with the research aims.

Ethical approval for the study was obtained from the Swedish Ethical Review Authority. All participants provided written informed consent prior to participation.

### Interview guide

A semi-structured interview guide was developed to address the research questions [21] (Additional file 2). Topics of the guide focused on participants’ perspectives of healthy aging, including factors that facilitate or hinder their ability to age as they desire. The guide comprised three main questions and five follow-up questions, along with prompts designed to encourage participants to share their experiences, views and thoughts on healthy aging. The interview guide was piloted with three older adults in a group interview and one individual interview conducted by two of the researchers (NF, JA), who were responsible for conducting the subsequent focus group interviews. Based on the pilot interviews, minor adjustments were made to the wording and order of the questions.

### Data generation

The focus groups were carried out face-to-face between December 2023 and January 2024. Three female researchers with backgrounds in public health (JA, AL) and physiotherapy (NF) and experienced in interviewing older adults facilitated the focus groups in pairs. One researcher conducted all focus group interviews (NF) and the other two alternated roles (JA, AL). The researchers had never met any of the participants prior to the study. The focus group interviews took place at the Aging Research Center of Karolinska Institutet, located 2.5 km from Kungsholmen. They were held in a quiet room near the entrance to ensure a comfortable environment for participants.

Upon arrival for the focus groups, participants received written information about the study in Swedish, as all participants were fluent in the language. Along with this, a consent form was provided. All participants were given the opportunity to ask any further questions about the study and what their involvement would mean.

The main questions in the interview guide were asked in all focus groups. One of the two researchers led the focus group interviews, while the other took notes and only interrupted for clarification. All focus groups were conducted in Swedish, audio-recorded, and transcribed verbatim by a third party. Each focus group interview lasted approximately 1.5 h.

### Data analysis

Our study employs Reflexive Thematic Analysis (RTA), which is grounded in a constructionist epistemology [17, 18]. In RTA, coding is understood as an active and reflexive process, shaped by the researcher’s theoretical position and interpretative engagement with the data. Unlike traditional reliability approaches, which are rooted in a positivist stance, RTA does not aim to establish reliability metrics or generalizability. Instead, rigor in RTA is demonstrated through transparency in the analytic process, depth of engagement with the data, and the coherence of the themes generated [17].

For the data analysis, the following six phases were applied: familiarization with data, generating initial codes, identifying themes, reviewing themes, defining and naming themes, and generating the report (Table 2) [17, 22]. Throughout the analytic process, iterative reflexive dialogue was used as a key strategy to deepen interpretation and enhance analytic rigor. This involved regular discussions within the research team, during which preliminary codes, emerging themes, and underlying assumptions were critically examined. All transcripts were managed and coded using NVivo 15 (QSR International) to support systematic data organization during the analytic process. All authors completed a reflexivity exercise to examine their positioning in relation to the data and its interpretation (Additional file 3).

**Table 2:** Description of data analysis steps

*Familiarization with data*: Transcriptions of the first round of focus group interviews were read carefully by all team members to get an overview of and familiarize themselves with the data and reflect on potential patterns of meaning. This informed the design of the second round of interviews.
*Generating initial codes*: All transcripts were read multiple times by three researchers who conducted the interviews (NF, JA, AL). An open, inductive coding approach was taken, without predefined codes. Each researcher actively engaged with the data to construct initial codes that captured features of interest across the data. The codes were then discussed and compared, and data relevant to each code were grouped for further analysis.
*Identifying themes*: The initial coding process involved ongoing discussions between researchers NF, JA and AL, comparing the specifics of what each code captured and reflecting on the potential meanings. The codes were then refined and grouped into preliminary themes through an iterative reflexive dialogue between NF, JA and AL. Three transcripts were coded individually by NF and AL. Their coding was compared, and some minor differences identified in the coding were discussed. Then NF coded the remaining four transcripts.
*Reviewing themes*: Preliminary themes were presented to the research team. The themes were reviewed and refined through dialogue with the broader research team. This step was repeated as all data were reviewed over again.
*Defining and naming themes*: The themes were defined and named through a dialogue within the entire research team. Each theme was accompanied by representative quotes for illustration and trustworthiness of the results provided.
*Producing the report*: The description, names, and quotes for each theme were reviewed by the research team. Interpretations of the findings were drawn jointly through a reflexive dialogue relating back to the purpose of the study and existing literature. Quotes to support the findings were translated into English through discussion between the researchers to make the translation as accurate as possible.

### Findings

In total, 34 older adults participated across seven focus group discussions. Participants ranged in age from 63 to 88 years and included both women and men, with a predominance of female participants. Educational backgrounds varied across groups, with most participants having completed university or high school education (Table 1).

**Fig. 1:**
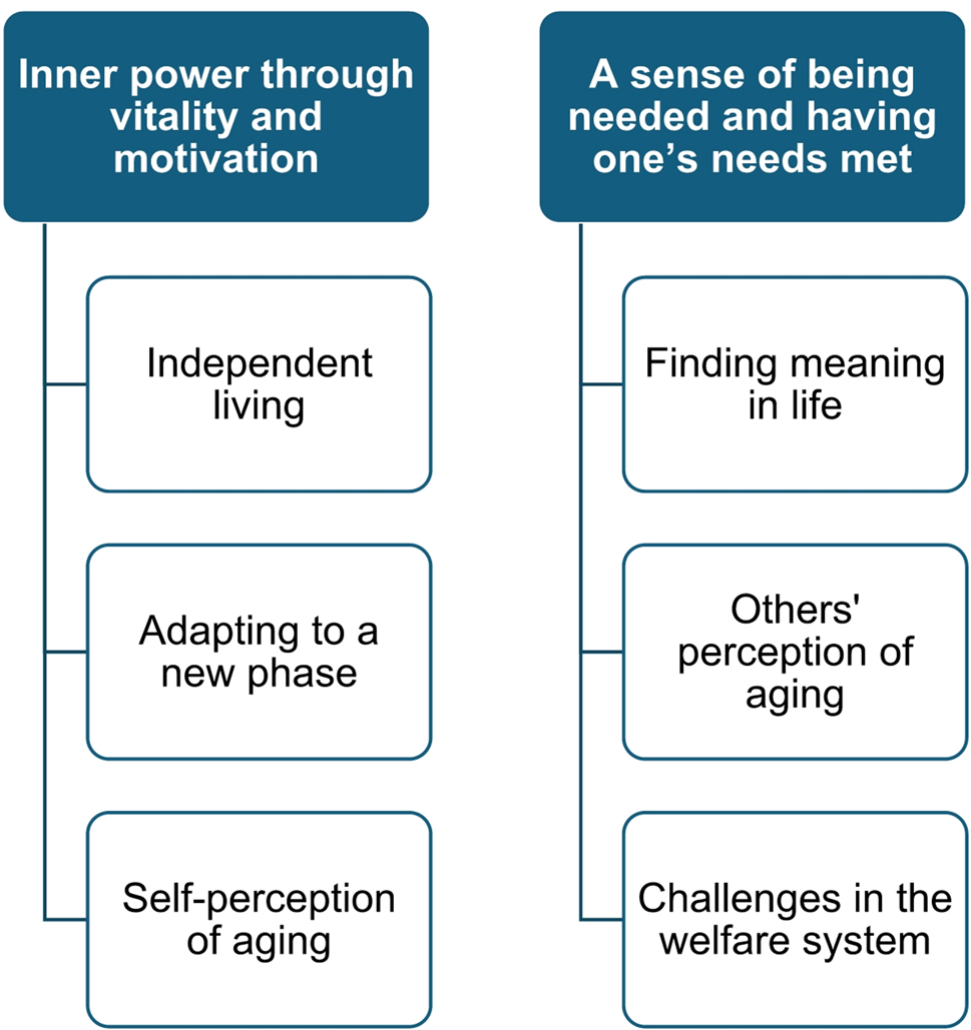
Themes and subthemes

The thematic analysis resulted in the construction of two overarching themes: “Inner power through vitality and motivation” and “A sense of being needed and having one’s needs met”. Each overarching theme is made up of subthemes that capture distinct but related aspects of a shared underlying concept presented in Fig. 1 , with detailed information provided in Additional file 4.

### Theme 1: inner power through vitality and motivation

The older participants experienced an inner drive that enabled them to handle obstacles that arise with aging. These obstacles might include physical ailments that made it harder to walk long distances or fatigue that prevented them from participating in activities they once enjoyed. This inner drive led the participants to act rather than simply accept their new circumstances. The participants described making a conscious effort to maintain their curiosity, recognizing its importance, and to explore new interests and hobbies. They also made sure to sustain their social connections by both meeting old friends and forming new relationships. The participants explained that they took responsibility for their aging process by staying physically active and participating in activities organized by associations targeting older adults. They described themselves as the main actors in their lives, willing to own their time. They viewed this new phase of life as a “second youth.”

#### Independent living

The participants emphasized that maintaining good health to manage daily activities and taking care of oneself was crucial. They also highlighted the importance of not becoming a burden to others. If assistance was needed, such as with cleaning, they expressed a preference for arranging it independently. Illness and inability to remain independent were seen as significant barriers to healthy aging.

*“The most important thing for me is to be able to take care of myself and avoid being a burden to others for as long as possible.”* (FG3).

The participants emphasized the importance of physical ability, particularly activities like walking, cycling, or gym exercise, but described bodily pain as a barrier to healthy aging. They acknowledged that physical ailments could be managed yet stressed that maintaining cognitive ability was even more crucial. Cognitive skills were seen as essential for staying self-aware, underscoring the importance of cognitive health over physical concerns.

*“Yes*,* as long as you can stay interested in what’s happening in the world and how it’s happening. I don’t think it’s too bad to sit there and not be able to move much if I can keep up with current events*,* keep up with what my children and grandchildren are doing. But if that gets narrowed down to the point where I have nothing left*,* then I wouldn’t want to go on.”* (FG5).

The participants highlighted the importance of adapting to digitalization and modernization and found it challenging to keep up with the pace of technological developments. They described how things used to be simpler, whereas nowadays it could take time to master new technical skills that moreover require constant updating. Modernization was not solely seen as a barrier by the informants but also as an asset.

*“Yes*,* I read my books on this [electronic device] because I can carry it in my bag. I mean*,* books come in different sizes*,* and I find it heavy to sit or lie in bed and read a thick book. This is great.”* (FG5).

The participants described their personal finances as crucial for healthy aging and expressed that their pensions were perceived as insufficient to cover their expenses. The gap between their income during employment and their pension after retirement was felt to be significant, limiting their opportunities to engage in various activities.

*“There is a significant drop in monthly income when transitioning from active employment to retirement*,* unless one has alternative solutions in place. Generally*,* it represents a substantial decrease*,* and if one is not prepared for this change*,* it can become challenging.”* (FG1).

Financial security was described as important to avoid feeling stressed about one’s housing, among others. Some participants expressed frustration over their economic situation, stating that they would need to actively protest, as the pension was too low. There were, however, some who noted that many activities organized by senior associations were often free.

#### Adapting to a new phase

The participants emphasized the importance of accepting the changes that aging brings with self-compassion and by setting the right expectations, including the challenges of growing older and frail. They acknowledged the uncertainty of the future, where sudden events could affect them or their loved ones. Learning to manage these challenges was seen as crucial, and they emphasized the need to cope with losses.

*“It can be difficult at times*,* but I believe it’s really important to be able to view yourself with humor and some emotional distance.”* (FG4).

*“I started playing padel a few years ago*,* and last summer I started playing golf. So*,* life has started to feel fun again; my husband passed away three years ago*,* so there’s been a lot to deal with. But there are still opportunities*,* even though illnesses can put a damper on things. You must find ways out*,* otherwise it just gets heavy. But when I talk with peers*,* it’s always about the body*,* and that can get in the way of a lot of things. But you must try to find joy in other areas.”* (FG2).

In this new phase of life, the participants needed to come to terms with the reality that life is not infinite. Frequent farewells and funerals for loved ones become regular reminders of this, requiring acceptance and adaptation.

*“That’s something I reacted to when my parents were my age*,* the way they talked about friends disappearing one by one. It’s something I couldn’t fully grasp when I was younger. But today*,* I’m in that situation myself*,* and I understand that feeling of life’s perimeter constantly shrinking.”* (FG2).

The participants acknowledged that it was sometimes difficult to accept the changes brought on by aging, which limited their ability to participate in activities they had enjoyed for most of their lives. They noted that reduced stamina made it impossible to participate in physical activities, such as hiking or skiing. Fatigue also hindered their ability to socialize with friends or attend small events, leading to feelings of sadness.

*“It’s becoming more frequent that people we know*,* friends*,* are struck by something because of aging. It happens more often the older you get*,* and it’s something that… yes*,* it needs to be handled in some way. Otherwise*,* I might fall back into melancholy.”* (FG4).

The participants explained that having a structured daily routine could help provide a sense of meaning and direction. They described the importance of continuing to have goals and milestones in life, with a structured week and weekend.

“*It’s easy to lose track of the difference between weekdays and weekends. That worries me a bit. When I lose track of what day it is*,* whether it’s Thursday or Tuesday*,* that’s when I agree that having goals and structure is important. Or even if it’s not cleaning or something like that*,* at least keeping track of what day it is.”* (FG1).

#### Self-perception of aging

The participants noted a disconnect between their chronological age and how they perceived themselves or how society views aging. They often felt younger than their actual age and didn’t identify with the label of “older adults.” They emphasized that mental age was important for healthy aging. It was crucial to continue living an active life without self-imposed limitations, regardless of growing older.

*“I have two sisters who are a bit younger than me*,* and we often talk about aging. We say we have three ages. First*,* there’s your chronological age on paper*,* then there’s your physical age*,* which you want to be younger than your actual age*,* and then there’s your mental age*,* which is really the most important.”* (FG4).

The participants highlighted the importance of focusing on the time left to enjoy life. They noted that there are opportunities to influence how one experiences aging by staying active and continuing to do the things they enjoy, even as they grow older. They also mentioned that spending time with people of different ages helped them feel younger themselves.

*“And if I’m in a group where everyone is older … then I see that they are older. I’d rather be in a mixed group. And I don’t see myself as older. That’s the strange part. Because I am—I see it in photos.”* (FG7).

### Theme 2: a sense of being needed and having one’s needs met

The participants described that when entering a new phase of life and no longer having the professional or family roles they were previously accustomed to, it becomes necessary to create new roles or find a new place where they feel needed and valued by those around them, as well as within society at large. They expressed that they now had the time to give back through their presence and involvement, which allows them to be significant to others. Additionally, they explained that the sense of being needed provides an internal motivation to remain active and enables them to positively influence those around them, fostering a supportive atmosphere among their peers. The participants also described the importance of having their own needs met in terms of services, access, and continuity in the welfare system.

#### Finding meaning in late life

The participants highlighted the importance of engaging in meaningful activities in older ages, maintaining a sense of purpose, and fostering social connections to support healthy aging, emphasizing the value of relationships, community engagement, and exploring new interests to combat loneliness and enrich later life.

*“Maybe try to prepare a little so that the rug isn’t pulled out from under you*,* leaving you standing there. But many people do things they didn’t have time for before. You can really indulge in… well*,* I don’t know but maybe take on longer projects that take a few hours each day or finally getting to try things you’ve been curious about but never had the time to do before.”* (FG1).

The participants highlighted the importance of maintaining a social network. Although these networks varied among the individuals, all emphasized the value of nurturing relationships, whether with a partner, friends, family, or grandchildren. They felt that relationships were essential for being part of a social context and avoiding loneliness. While making new friends through activities could be enriching, some also mentioned rekindling old friendships, such as reconnecting with former classmates to share memories. However, they also acknowledged that making new friends in later life may be challenging. Some participants reported becoming widowed, but also described subsequently forming a new romantic relationship. They emphasized the significance of these relationships in discovering new interests and enjoying fun activities together.

*“I was widowed when I was 61. Then I lived alone for nine years. And then I reconnected with an old friend*,* we met again—he was divorced. So*,* we have a lot of fun together*,* and it’s great. We ride motorcycles and do all sorts of fun things*,* things I never thought I’d be doing [laughs]. But it means so much*,* and I met him just when my grandchildren started school.”* (FG4).

#### Others’ perception of aging

The participants described a sense of insecurity and unease in society, where respect for older adults was perceived as low. They felt that they were often seen as a burden—taking up too much time and costing society—rather than being valued for their experience and wisdom. Many expressed frustrations over being overlooked or dismissed, contributing to a broader feeling of vulnerability. This lack of consideration was particularly evident in everyday situations, where they struggled to navigate a society that seemed structured for a faster pace, leaving them feeling marginalized and disrespected.

*“The old meaning of respect*,* valuing older adults for their experience and wisdom*,* isn’t very present in our culture. It often feels like they’re just in the way. Of course*,* there are kind people and others who provide a counterbalance. But there’s also a sense of not being able to just relax and allow an older person the time they need.”* (FG1).

*“Yes*,* in other cultures*,* there is a completely different approach. Older adults are really valued there.”* (FG3).

The participants emphasized how time could work against them. They noted that society was not structured to accommodate the extra time they needed as their pace slows down. Tasks like boarding a bus with a walker or entering a PIN code now took longer, and they felt discriminated against and disrespected because of their age.

*“But sometimes*,* I feel like there’s a need to just let an older person take their time—whether it’s swiping a card or entering a code—without rushing or sighing in impatience. I’m still quick at paying*,* but it’s about allowing the time to be taken…”* (FG1).

The participants voiced concerns about rising crime rates in society, describing feelings of vulnerability and uncertainty about their ability to protect themselves. On the other hand, they also noted certain people’s sensitivity and humanity towards older adults.

*“Yes*,* but on the other hand*,* I am often pleasantly surprised. This may not be seen as an expression of respect for older adults*,* but when I use public transportation*,* I always stand. It is very common for people of varying ages to say*,* ‘Yes*,* please*,* take my seat.”* (FG3).

#### Challenges in the welfare system

The participants identified changes in the healthcare system and the perceived shortage of nursing homes and home care services as significant barriers to healthy aging, particularly as the aging population continues to grow.

*“I realize that it’s becoming harder and harder to get a good nursing home because there are more and more of us…”* (FG4).

Participants expressed significant concern that healthcare services may not be functioning as they should today, highlighting issues related to access, continuity, and the potential lack of sufficient services for older adults. Many perceived the healthcare system as a growing societal problem. A particular worry was that it was no longer as easy to contact their general practitioner, and the sense of security in being able to visit the same doctor regularly had disappeared.

*“Part of that is having good healthcare—having a good family doctor or whatever it may be—someone you trust and are satisfied with. You might not call them every day to check if you’re healthy*,* but it’s comforting to know you have check-ups and can expect support. I find that difficult to find; it’s not easy.”* (FG1).

Participants also emphasized the importance of feeling safe and having control over their healthcare. They described how it was crucial to know that there was support to rely on, but that it felt more difficult to obtain this sense of security in the current system. The increased reliance on automated systems and digital services, such as robots and chat functions, was seen as an obstacle to personal contact. Many missed human interactions and found it difficult to receive help through these more impersonal digital channels.

In addition, participants pointed out deficiencies in eldercare, where there were sometimes long intervals between home care visits, leading to a perception that older adults did not receive the necessary care in a timely manner. These shortcomings contributed to an unfavorable aging process, where appropriate care and contact were not always available when needed most.

*“What you’re describing*,* I believe all of us here are thinking about this*,* how things are in eldercare. And we will most likely all become dependent on it at some point. I think this is something each of us can feel a certain concern about—what will it look like when we are alone and aging.”* (FG2).

## Discussion

We constructed two key themes shaping older adults’ experiences of healthy aging, namely their inner power and motivation as well as their feelings of being needed and having their needs met. The findings reflect the participants’ perspectives on the importance of maintaining autonomy, adapting to life transitions, fostering social connections, and navigating societal structures that impact healthy aging.

Participants’ reflections on the relevance of inner power and optimism in remaining both physically and cognitively independent align with existing Swedish research, which reports that many older adults actively manage their health [23]. However, actively managing one’s own health might be challenged by societal structures and expectations. Indeed, the roles of older adults in relation to intergenerational dependencies and the provision of informal care vary across different communities and families [24, 25], potentially restricting the individual’s time for undertaking activities of one’s own interests. Recent research from European high-income countries has shown that people from lower socioeconomic groups are more likely to take responsibility in providing informal care compared to people from higher socioeconomic groups [26]. Sweden is no exception, where lower socioeconomic position rather than cultural differences has been associated with providing informal care to a relative [27]. Based on the current study’s focus group discussions, participants seemed to balance providing informal care and fulfilling oneself, possibly reflecting middle-class status. Furthermore, beyond an individual’s capabilities and socioeconomic position, as well as social and environmental contexts, the ability to maintain health and well-being and shape one’s own life is also constrained by coping capacity and power relations [28]. In this context, power relations refer to older adults’ experiences of perceived dependence, limited control over decisions, and asymmetries of authority within health and social care systems, as discussed in previous Swedish studies [23]. Taken together, several of the issues raised by participants, such as opportunities for social participation, independent living, mobility, access to services, and challenges related to digitalization, also resonate with domains described in the WHO Age-Friendly Cities Framework, which emphasizes the role of physical, social, and service environments in shaping older adults’ everyday lives [29].

Moreover, participants shared their perspectives on being older citizens, expressing concerns that ranged from feeling a lack of respect and value to shortcomings in the healthcare system, such as difficulties in consistently seeing the same primary care physician. Another concern involved uncertainty about securing a place in a preferred care home when the need arises. These reflections underscore that Sweden is not immune to ageist attitudes or insufficient investment in the fastest-growing segment of the population. In addition, modernization of essential services may introduce new forms of exclusion. Digital exclusion remains a substantial barrier for many older adults; for example, approximately 18% of people aged 65 years and older do not use the internet for personal purposes or lack access altogether [30]. As services such as banking and healthcare increasingly shift to digital platforms, this may force reliance on family members or friends, potentially undermining older adults’ sense of independence, autonomy, and self.

Whilst participants often reported feeling younger than their chronological age and described leading physically and socially active lives, they also referred to friends and relatives whose freedom was limited by factors such as immobility. These reflections highlight the heterogeneity of older age and relate to the development of the concepts of the third age [31] and, later, the fourth age [32], which were introduced to counteract the marginalization of older adults. This distinction emphasizes individual capacity rather than chronological age: the third age is characterized by individuals’ potential to achieve personal fulfillment and actively participate in society, whereas the fourth age is often marked by increased dependency. However, despite its good intentions, this conceptual division has been criticized for exacerbating ageism toward those in the fourth age [33].

In addition to the health heterogeneity of later life, participants also described a sense of dynamism through changing preferences over time. Rather than striving to maintain former activities, they emphasized adapting their goals and interests in response to physical limitations, loss, and a heightened awareness of life’s finitude. This included engaging in new activities, finding alternative sources of enjoyment, and prioritizing everyday structure and emotionally meaningful pursuits as ways of coping with uncertainty. This aligns with recent research suggesting that individuals’ preferences shift as their perceived time horizon shrinks [34]. Carstensen and Reynolds (2023) argue that in later life, people increasingly prioritize actions that offer immediate emotional satisfaction over those with long-term benefits. The present findings support this perspective, as participants reported engaging in activities that brought joy and pleasure, such as playing sports. Additionally, the findings contribute to a growing body of research on the impact of a shortened time horizon [35], as participants acknowledged future uncertainty and emphasized the importance of learning to cope with loss and change.

This study contributes to a deeper understanding of health beyond the mere absence of disease by illustrating how the WHO Healthy Ageing Framework aligns with older adults’ own perceptions and preferences, thereby demonstrating its relevance in real-world contexts [10]. Specifically, the findings extend the Framework by highlighting the role of inner motivation: despite experiencing reduced functional abilities, participants remained motivated to live independently, often supported by self-compassionate attitudes and enabling physical and social environments. Viewed in this way, the Age Friendly Cities Framework [29] may offer a useful complementary perspective for interpreting how structural and environmental factors enable or constrain older adults’ agency in later life. This is a particularly important implication given the recognized need for actively including older adults’ voices in research using this WHO Healthy Ageing Framework, to more effectively address their actual needs ([9, 12–13]). These insights can guide policy and efforts aimed at fostering supportive conditions and environments that empower individuals to lead fulfilling lives, while preserving their autonomy to determine what is practically achievable—an idea central to the concept of individual capabilities within the WHO Healthy Ageing Framework [10].

### Study limitations and strengths

Study limitations include selection bias and participant homogeneity, as participants were part of an aging cohort study (SNAC-K) and resided in an advantaged urban area. In addition, older adults from minority ethnic groups were underrepresented, reflecting a broader limitation of the SNAC-K cohort that may limit transferability. From an equity perspective, the relatively advantaged socioeconomic context of the study population may restrict insights into the barriers to healthy aging experienced by older adults living in more disadvantaged circumstances. The findings can therefore be interpreted as reflecting a *best-case scenario* of how healthy aging is experienced within a well-resourced welfare state context. Furthermore, SNAC-K participants who did not attend the proband day could not enroll in the study, potentially excluding individuals living in care homes or those with cognitive or mobility impairments that prevented attendance. The researchers also acknowledge their subjectivity throughout the analysis and interpretation of findings [18]. As professionals with experience in health and aging, our initial understanding was shaped by prior engagement with the research area. While this pre-understanding informed our approach, we entered the analysis with curiosity and openness, aiming to explore how these perspectives might manifest in a Swedish context. The interplay between fresh viewpoints from some team members and the prior insights of others fostered rich and reflexive dialogue throughout the analytic process [18]. In addition, the findings were not returned to participants for feedback or verification. However, there is limited evidence that this practice enhances the validity of qualitative research findings [36]. The focus group methodology may also have attracted participants who were comfortable sharing their views in a group setting or who were more socially connected, potentially limiting the inclusion of more socially isolated experiences [16]. Moreover, data collection was conducted during the winter months, which may have influenced participants’ opportunities for outdoor physical and social activities. Seasonal variation in weather and daylight may affect how and where older adults engage in everyday activities and should therefore be considered when interpreting the findings [37].

Strengths of the study include the transparent and detailed reporting of the research process and the analytical approach, involving multiple researchers in the planning, analysis, and discussion of the findings. Trustworthiness was sought through reflecting on our role as researchers and through a clear description of how the data were analyzed. Efforts were made to enhance transferability of the study to other contexts by describing the participants, setting, and phenomena studied [38]. An additional strength of the focus group methodology was the group dynamic, which may have facilitated richer discussions through shared lived experiences and yielded deeper insights than individual interviews.

## Conclusions

In conclusion, this study suggests that older adults without significant health issues who reside in relatively affluent urban areas can draw on their inner motivation to shape and lead meaningful lives. The findings emphasize the urgent need to promote more equitable opportunities for healthy aging for all older adults. Greater societal investment in older adults would not only affirm their value but could also help counteract ageism and reduce the commonly reported feeling of being a burden to society. Furthermore, the influence of the physical and social milieu on participants’ inner motivation underscores the importance of recognizing the environment as an integral component of health—a perspective that is often-overlooked yet central to the WHO Healthy Ageing Framework. Further research on the topic should be undertaken in other countries and in populations of more socially deprived areas to enrich our understanding of how the physical and social milieu influence inner motivation and healthy aging. 

## Supplementary Information

Supplementary Material 1.

## Data Availability

The data generated and/or analyzed in the current study are not publicly available due to data protection and anonymization requirements but are available from the corresponding author on reasonable request.
