# Supplementary material for: Perceptions on healthy aging: insights from focus group interviews of older adults in Sweden
Source: Arch Public Health. 2026 Apr 16;84:78. doi: 10.1186/s13690-026-01915-1 (PMC13085716; doi:10.1186/s13690-026-01915-1)
Supplement: Supplementary file 1 — Supplementary Material 1. [file 13690_2026_1915_MOESM1_ESM.docx]

**Additional file 1.** Standards for Reporting Qualitative Research.

|  | **Standards for Reporting Qualitative Research (SRQR)*** |  |
| --- | --- | --- |
|  | <http://www.equator-network.org/reporting-guidelines/srqr/> |  |
|  |  | **Page/line no(s).** |
| **Title and abstract** | |  |
|  | **Title** - Concise description of the nature and topic of the study Identifying the study as qualitative or indicating the approach (e.g., ethnography, grounded theory) or data collection methods (e.g., interview, focus group) is recommended | Page 1 |
|  | **Abstract** - Summary of key elements of the study using the abstract format of the intended publication; typically includes background, purpose, methods, results, and conclusions | Page 2 |
|  |  |  |
| **Introduction** | |  |
|  | **Problem formulation** - Description and significance of the problem/phenomenon studied; review of relevant theory and empirical work; problem statement | Page 4 |
|  | **Purpose or research questio**n - Purpose of the study and specific objectives or questions | Page 5 |
|  |  |  |
| **Methods** | |  |
|  | **Qualitative approach and research paradigm** - Qualitative approach (e.g., ethnography, grounded theory, case study, phenomenology, narrative research) and guiding theory if appropriate; identifying the research paradigm (e.g., postpositivist, constructivist/ interpretivist) is also recommended; rationale** | Page 6 |
|  | **Researcher characteristics and reflexivity** - Researchers’ characteristics that may influence the research, including personal attributes, qualifications/experience, relationship with participants, assumptions, and/or presuppositions; potential or actual interaction between researchers’ characteristics and the research questions, approach, methods, results, and/or transferability | Page 9, 22 |
|  | **Context** - Setting/site and salient contextual factors; rationale** | Page 6 |
|  | **Sampling strategy** - How and why research participants, documents, or events were selected; criteria for deciding when no further sampling was necessary (e.g., sampling saturation); rationale** | Pages 5-6 |
|  | **Ethical issues pertaining to human subjects** - Documentation of approval by an appropriate ethics review board and participant consent, or explanation for lack thereof; other confidentiality and data security issues | Page 7 |
|  | **Data collection methods** - Types of data collected; details of data collection procedures including (as appropriate) start and stop dates of data collection and analysis, iterative process, triangulation of sources/methods, and modification of procedures in response to evolving study findings; rationale** | Pages 7-8 |
|  | **Data collection instruments and technologies** - Description of instruments (e.g., interview guides, questionnaires) and devices (e.g., audio recorders) used for data collection; if/how the instrument(s) changed over the course of the study | Page 8-9 |
|  | **Units of study** - Number and relevant characteristics of participants, documents, or events included in the study; level of participation (could be reported in results) | Page 9 |
|  | **Data processing** - Methods for processing data prior to and during analysis, including transcription, data entry, data management and security, verification of data integrity, data coding, and anonymization/de-identification of excerpts | Page 8-9 |
|  | **Data analysis** - Process by which inferences, themes, etc., were identified and developed, including the researchers involved in data analysis; usually references a specific paradigm or approach; rationale** | Page 9 |
|  | **Techniques to enhance trustworthiness** - Techniques to enhance trustworthiness and credibility of data analysis (e.g., member checking, audit trail, triangulation); rationale** | Page 9, 22 |
|  |  |  |
| **Results/findings** | |  |
|  | **Synthesis and interpretation** - Main findings (e.g., interpretations, inferences, and themes); might include development of a theory or model, or integration with prior research or theory | Pages 9-18 |
|  | **Links to empirical data** - Evidence (e.g., quotes, field notes, text excerpts, photographs) to substantiate analytic findings | Pages 9-18 |
|  |  |  |
| **Discussion** | |  |
|  | **Integration with prior work, implications, transferability, and contribution(s) to the field -** Short summary of main findings; explanation of how findings and conclusions connect to, support, elaborate on, or challenge conclusions of earlier scholarship; discussion of scope of application/generalizability; identification of unique contribution(s) to scholarship in a discipline or field | Pages 18-21 |
|  | **Limitations** - Trustworthiness and limitations of findings | Page 21-22 |
|  |  |  |
| **Other** | |  |
|  | **Conflicts of interest** - Potential sources of influence or perceived influence on study conduct and conclusions; how these were managed | Page 24 |
|  | **Funding** - Sources of funding and other support; role of funders in data collection, interpretation, and reporting | Page 23-24 |
|  |  |  |
|  | *The authors created the SRQR by searching the literature to identify guidelines, reporting standards, and critical appraisal criteria for qualitative research; reviewing the reference lists of retrieved sources; and contacting experts to gain feedback. The SRQR aims to improve the transparency of all aspects of qualitative research by providing clear standards for reporting qualitative research. |  |
|  |  |  |
|  | **The rationale should briefly discuss the justification for choosing that theory, approach, method, or technique rather than other options available, the assumptions and limitations implicit in those choices, and how those choices influence study conclusions and transferability. As appropriate, the rationale for several items might be discussed together. |  |
|  |  |  |
|  | **Reference:** |  |
|  | O'Brien BC, Harris IB, Beckman TJ, Reed DA, Cook DA. **Standards for reporting qualitative research: a synthesis of recommendations.** *Academic Medicine*, Vol. 89, No. 9 / Sept 2014  DOI: 10.1097/ACM.0000000000000388 |  |

**Additional file 2.** Semi-structured interview guide.

**Semi-structured interview guide for focus group interviews**

Thank you very much for coming here today! My name is XXX and I will be leading this interview, and this is XXX, who will be acting as an observer (and thus taking some notes) throughout the interview.

The purpose of our project is to learn about people's experiences of aging, what is important during that process and how to imagine your future when you become old. We also want to address how participants in the SNAC-K study experience being part of the study, and what you think about the information being collected.

We have invited you here today to hear your experiences and thoughts on the matter. I'm going to ask a few questions and then you can add more at the end of the interview if anything comes up.

- Provide the informed consent document.
- Explain the importance and authorization to record the interview, insisting on the confidentiality of the information.
- Inform about the pseudonymized document that will be used for collecting sociodemographic data as well as the participation time in the SNAC-K study.
- Participants’ answers are followed up with questions such as: Do you wish to tell me more about this? What is this due to? What is missing? What is needed?

**PART I: PEOPLE'S EXPERIENCES WITH AGING**

1. What does healthy aging mean to you? (feel free to take a couple of minutes to think about this question)

NOTE: if they do not respond spontaneously, you may personalize the question to their own cases: *How would you like to age? How would you like your old age to look like from now on?*

1. What do you think it takes to be able to age the way you want?

NOTE I: if they do not respond spontaneously, you may relate this question to question 1: *Of the aspects that you have mentioned that influence healthy aging, which would you consider the most important and why?*

NOTE II: by “aspects” we mean help, tools, strategies of any kind, personal, family- or community-related, etc.

1. What possible obstacles prevent you from aging the way you want?

NOTE I: if they do not respond spontaneously, you may relate this question to question 1: *Of the aspects that you have mentioned that influence healthy aging, do you personally think you have such resources? Which ones do you miss most?*

NOTE II: by obstacles we mean difficulties that may be encountered of all kinds, personal, family-related, social, architectural, economic, technology or digitalization-related, the Covid pandemic, etc.

**PART II: SNAC-K PARTICIPANTS’ EXPERIENCE BEING PART OF THE STUDY**

1. How do you see the SNAC-K study of which you are part?

NOTE: if they do not respond spontaneously, you may ask: *Why did you join the study? What are the main reasons why you continue? Do you think other participants’ experiences are similar to yours?*

1. Do you think that the SNAC-K study satisfactorily captures those dimensions or aspects that we discussed earlier about how we understand healthy aging? Why or why not?

NOTE: additional related questions: *Which of those aspects are collected satisfactorily and which ones are missing or could be collected better?*

1. Do you have any other thoughts that you want to highlight?

**END OF INTERVIEW**

- Finish by thanking them again for their participation, asking how they felt and informing them about our intention to contact them again to send them a draft of the provisional results (by email).
- The moderator and the observer will meet once the participants leave to fill in the notebook with their impressions about the focus group (e.g. level of participation, possible preliminary findings, what was expected to be discussed and did not arise and vice versa).

**Semistrukturerad intervjuguide för fokusgruppsintervjuer**

Tack så mycket för att ni kom hit idag! Jag heter XXX och jag kommer att leda den här intervjun, och det här är XXX, som kommer att agera som observatör (och ta anteckningar) under intervjun.

Syftet med det här projektet är att vi vill lära oss om människors upplevelser av åldrandet, vad som är viktigt under den processen och hur man föreställer sig sin framtid när man blir gammal. Vi vill också ta upp hur deltagare i SNAC-K-studien upplever att vara en del av studien, och vad ni tycker om informationen som samlas in.

Vi har bjudit in er hit idag för att höra era erfarenheter och tankar om detta. Jag kommer att ställa några frågor och sedan kan ni lägga till fler i slutet av intervjun om något dyker upp.

- Tillhandahåll dokumentet för informerat samtycke.

- Förklara vikten och godkännandet av att spela in intervjun och förtydliga att informationen är konfidentiell.

- Informera om det pseudonymiserade dokumentet som kommer att användas för insamling av sociodemografisk data samt deltagandetiden i SNAC-K-studien.

– Deltagarnas svar följs upp med frågor som: Vill du berätta mer om detta? Vad beror detta på? Vad saknas? Vad behövs?

**DEL I: MÄNNISKORS ERFARENHETER AV ÅLDRANDE**

1. Vad betyder hälsosamt åldrande för dig? (ta gärna ett par minuter att fundera över denna fråga)

OBS: om de inte svarar spontant kan du anpassa frågan till deras egna fall: Hur skulle du vilja åldras? Hur skulle du vilja att din ålderdom skulle se ut från och med nu?

1. Vad tror du att det krävs för att kunna åldras som du vill?

OBS I: om de inte svarar spontant kan du relatera denna fråga till fråga 1: Av de aspekter som du har nämnt som påverkar hälsosamt åldrande, vilken skulle du anse vara viktigast och varför? OBS II: med "aspekter" menar vi hjälp, verktyg, strategier av alla slag, personliga, familje- eller samhällsrelaterade, etc.

1. Vilka eventuella hinder skulle kunna hindra dig från att åldras som du vill?

OBS I: om de inte svarar spontant kan du relatera denna fråga till fråga 1: Av de aspekter som du har nämnt som påverkar hälsosamt åldrande, tror du att du har sådana resurser? Om inte, vilka saknar du mest?

OBS II: med hinder menar vi svårigheter som kan uppstå av alla slag, personliga, familjerelaterade, sociala, arkitektoniska, ekonomiska, teknologiska eller digitaliseringsrelaterade, Covid-pandemin, etc.

**DEL II: SNAC-K-DELTAGARES ERFARENHET ATT ÄR EN DEL AV STUDIEN**

1. Hur ser du på SNAC-K-studien som du är en del av?

OBS: om de inte svarar spontant kan du fråga: Varför gick du med i studien? Vilka är de främsta anledningarna till att du fortsätter? Tror du att andra deltagares erfarenheter liknar dina?

1. Anser du att SNAC-K-studien fångar de dimensioner eller aspekter som vi diskuterade tidigare om hur vi förstår hälsosamt åldrande? Varför eller varför inte?

OBS: ytterligare relaterade frågor: Vilka av dessa aspekter samlas in på ett tillfredsställande sätt och vilka saknas eller skulle kunna samlas in bättre?

1. Har du några andra tankar som du vill lyfta fram?

**SLUT PÅ INTERVJU**N

- Avsluta med att tacka dem igen för deras deltagande, fråga hur de upplevde intervjun och informera dem om vår avsikt att kontakta dem igen för att skicka dem ett utkast till de preliminära resultaten (via e-post).

- Moderatorn och observatören kommer att träffas när deltagarna lämnar lokalen för att fylla i anteckningsboken med sina intryck av fokusgruppen (t.ex. deltagandegrad, möjliga preliminära resultat, vad som förväntades diskuteras och inte kom upp och vice versa).

**Additional file 3.** Reflexivity exercise.

The following table is built inspired by the book of Braun & Clarke ‘Thematic analysis, a practical guide’ 2022.

| Personal reflexivity | | | | | | | |
| --- | --- | --- | --- | --- | --- | --- | --- |
| Positioning |  | MK | NF | AKW | AL | ACL | JA |
| Consider where you occupy positions of social privilege. |  | White, highly educated | White | White | White | White, upper middle class, highly educated, European citizen, economically, socially and functionally independent | White |
| Consider where you occupy positions of social marginality. |  | Female, immigrant, non-native speaker of Swedish | Female | Female | Female | Female, regular immigrant | Female, immigrant |
| How does your personal background shape the way you engage in our study? |  | Gender, socioeconomic class | Middle class | Middle class | Middle class | Commitment to scientific rigor and evidence-based care provision | As a public health researcher with experience in studying how healthcare systems are organised, I was particularly attentive to participants’ reflections on the role of health and social care system for health aging. At the same time, conversations with older family members had me reflect more on everyday meanings of healthy aging. I tried to avoid prescriptive assumptions by staying close to participants’ experiences during coding and team discussions. |
| How do your political and ideological commitments reflect on the way you see our research or our data? |  | Socialism, secularity, but respectful to all religions | I affirm the inherent equal value of every human being and their fundamental right to express their views and have them heard. | I affirm the inherent equal value of every human being and their fundamental right to express their views and have them heard. | I affirm the inherent equal value of every human being and their fundamental right to express their views and have them heard. | Adherence and respect for democratic and socialist values, along with human and civil rights, in both political and professional spheres. | My prior research has focused on equity and universal access in health and social care, which may incline me to foreground structural barriers and service-related issues in the data. However, the team’s varied disciplinary perspectives acted as a reflexive check, helping to ensure that interpretations did not over-focus on system-level explanations. |
|  | | | | | | | |
| How are your research training and your experiences? |  | 2 years master’s degree, 4 years PhD studies | I’m a licensed physiotherapist | I’m a licensed physiotherapist | Public health professional | Double university degree, master’s, PhD, postdoc at different universities in Spain, France and Sweden.  Main focus on quantitative research, with strong interest in mixed methods. | I am a PhD-level public health researcher with experience in both quantitative and qualitative methods. |
| What type of institution do you work for? How is that relevant? |  | Public academic institution | I’m conducting research at a university in Sweden | I’m conducting research at a university | Researcher at a university | European public university, with a strong biomedical orientation towards the understanding of health and well-being. | Karolinska Institutet (medical university), Department of Neurobiology, Care Sciences and Society, with training in Public Health. This positioning bridges biomedical/clinical and public health perspectives, shaping my attention to both functional health and the wider social and system context. |
| What methods do you use? |  | Quantitative and qualitative methods | Both qualitative and quantitative methods | Both qualitative and quantitative | Qualitative and quantitative methods | Quantitative (95%).  Some experience with thematic and content analysis. | Both qualitative and quantitative |
| What theoretical assumptions or commitments do you have related to qualitative research? |  | See it as useful for determining lived experiences | No assumptions | No assumptions | I consider qualitative research very important | Positivism in my mind (shaped by my training), constructivism in my heart (driven by my personal growth). | From a critical realist perspective, I assume healthy aging is shaped by real social and material conditions, while recognising that individuals experience and interpret these conditions differently. |
| Consider your fears and hopes about your research. |  | I hope it’s relevant and useful | I aim to develop a deeper understanding in different research areas and topics. | I aim to develop a deeper understanding of diverse phenomena and the experiences of individuals. | Fears include not being enough open-minded. Hopes include contributing to society | My priority is to provide insightful results, relevant to the current public health debate. | Hope to produce an interpretive RTA that does justice to the participants. Fear becoming too descriptive or just confirming preconceived ideas (e.g., WHO alignment), or missing important nuances in the material. |
|  | | | | | | | |
| How are your positionings or life experiences related to your research topic? |  | I am in my early 30s, so I do not relate to our participants’ experiences yet | My experience as a physiotherapist and a researcher in aging is closely related to this topic. | My experience as a researcher in aging and a physiotherapist is closely related to this topic. | I have a strong and genuine interest in aging, health promotion and disease prevention | As a geriatric epidemiologist, I have gained an increasing understanding of the complexity of health in old age, along with the significant challenges it presents, not only for research but also for the care of older individuals. These are precisely some of the aspects we explore in this work. | I am professionally close to the topic through many years of doing research within social gerontology at the Aging Research Center and from previous work in home help services with older adults. |
| What assumptions to you have around the topic? |  | I assume all people want to age well and they should be able to do so | I assume healthy aging is influenced by several factors such as social, psychological, and biological factors. The experiences vary between individuals. | I assume healthy aging is influenced by several factors such as physiological and social factors, and experiences vary between individuals. | I assume aging is influenced by multiple individual and societal factors. | I fully believe in the multidimensional nature of the determinants of healthy aging, as well as in the need to understand health beyond the mere absence of disease. | I assume healthy aging is multidimensional and shaped by both individual resources and social conditions, and that experiences vary substantially between individuals and are patterned by inequities. I also assumed that understandings and priorities around healthy aging would differ across later life stages, with “younger-old” and “older-old” adults emphasising different aspects. |
| How might your participants perceive you? |  | I have not interacted with participants directly | As knowledgeable and professional | I was not directly involved in the interviews | Friendly, a good listener, professional | I was not directly involved in the interviews | Welcoming, friendly, and genuinely interested in learning from participants’ experiences. |
| Which positions of privilege or marginality do you occupy in relation to your topic and your participants? |  | Highly educated, young, relatively healthy | Highly educated and relatively young. | I have educational and professional privilege. | I have educational and professional privilege. | Being a researcher in geriatric epidemiology could have affected the freedom and spontaneity of speech among participants, had I been directly involved in the interviews. | Mid-life and highly educated, I occupy educational/professional privilege compared with most participants. I have some personal experience of health problems, but not to the extent of participants’ age-related conditions, so I remain largely an outsider to their lived experience. |
| Are you and insider or an outsider researcher? Or both? How does this shape your research methods and your relationships with the participants? Any advantages or risks? |  | As an outsider, I have a different perspective and an open mind to our findings | I am both: insider in professional knowledge, outsider in personal experiences. I have an open mind to findings. | I am both: insider in professional knowledge, outsider in personal experiences. This helps balance understanding and objectivity but may risk misinterpretation. | Primarily an outsider as I am middle-aged yet very familiar to the situation of the participants, which facilitates understanding | Being an outsider may be positive from an impartiality perspective, but it could also create a distance between the content and my interpretation of it. | Both: insider professionally (aging research; experience in home help services) and outsider experientially (mid-life, with fewer age-related limitations). Advantage: contextual understanding and ability to ask relevant follow-up questions. Risk: relying too much on pre-understandings or being perceived as an authority, which could shape what participants chose to share |
| Other factors, positionings or experiences that you find meaningful in your relation to our research? |  | I have aging parents, and I am familiar with their experiences | I have aging parents. I am familiar with their experiences and perspectives. | My commitment to equality and valuing participants’ perspectives, and interdisciplinary background, informs my approach. | My aging parents and uncles are of lower socioeconomic background with little education which provides me insight. | My little experience with the applied analytical techniques makes me feel somewhat insecure. | Supporting my grandmother near the end of life have reinforced my understanding of the importance of preserving autonomy as physical abilities diminish, and highlighted that quality of life is highly individual and may shift with age and changing health status. |

**Additional file 4.** Detailed description of themes, subthemes, codes and text they were generated from.

| **Theme** | **Subtheme** | **Code** | **Code description** | **Verbatim (translated from Swedish)** |
| --- | --- | --- | --- | --- |
| **Inner power through vitality and motivation** | Independent living | Not be a burden | Maintaining the ability to care for oneself is essential to preserving independence and minimizing reliance on others. | *"The most important thing for me is to be able to take care of myself and avoid being a burden to others for as long as possible."* (Focus group interview 3) |
|  |  | Stay healthy physically, cognitively, and mentally | Maintaining mobility and preserving both physical and cognitive functioning are critical components of perceived health and well-being. | *"That was probably the most important thing I forgot to mention: health. Both physical and mental health."* (Focus group interview 4) |
|  |  | Digitalization and modernization | Modern society presents challenges due to rapid social transformations and the increasing need to navigate digital technologies in order to maintain independence. | *"…Personally, I find all the digital aspects very difficult to absorb and learn. I constantly must ask for help from my colleagues, my daughter, and others around me. This creates a barrier for me, as there are so many digital tools in use today."* (Focus group interview 1) |
|  |  | Personal finances | Financial security is essential for reducing stress related to housing stability and for enabling participation in meaningful social and recreational activities | *"Being in reasonably good health and having a stable financial situation where one doesn’t constantly need to worry about 'How will I make it through this month?' I believe that kind of struggle is incredibly difficult, and far too many people are facing it today."* (Focus group interview 3) |
|  | Adapting to a new phase | Accept and act | Successful adaptation to a new life phase requires acceptance of, and alignment with, changing circumstances. | *“It takes time to find a good rhythm with retirement life; it took me a couple of years.”* (Focus group interview 1) |
|  |  | Humor and distance | Coping with the changes associated with aging often requires the use of humor and the ability to maintain perspective. | *"Yes, I completely agree, and I find it a bit difficult at times, but I believe it's incredibly important to be able to look at oneself with perspective and humor.”* (Focus group interview 4) |
|  |  | Barriers | Entering a new phase of life entails a range of challenges that must be effectively managed. | "However, I feel that many relatives and friends have passed away. There are not many left. That's just the way it is." (Focus group interview 7) |
|  |  | Melancholy | Age-related changes can be difficult to accept or adapt to, particularly when accompanied by increased social isolation and reduced ability to engage in previously enjoyed activities. | *"…But I probably wasn't prepared to find myself in those moments of melancholy from time to time, trying, of course, to accept it. This is just how it is, and the alternative would be worse. I’ve had a good life and still do, but even so, I find myself feeling that melancholy. I sometimes find that quite heavy to bear."* (Focus group interview 4) |
|  |  | Structure | A structured daily routine provides a sense of purpose and facilitates goal attainment by supporting focus and progress toward intermediate objectives. | *"You have goals when you work. I believe that, for me, it is beneficial to have a structure, a routine, and a weekend, along with goals that I strive to achieve."* (Focus group interview 1) |
|  | Self-perceptions of aging | One’s own experiences of aging | An individual’s perception of aging is influenced by their mindset and the extent to which they emphasize chronological age versus subjective psychological well-being. | *”I can't get it! I turned 80 in April. I can't accept or even relate to that number at all.”* (Focus group interview 1) |
|  |  | Time left to enjoy | Even in advanced age, individuals may still have the opportunity to pursue unfulfilled goals or engage in new experiences. | *“…Not focusing on aging but still feeling young in your heart. You keep living as before, with the added freedom of owning your time. You can spend time with whomever you like and do what you want. It's wonderful. It feels great to finally do what you might have dreamed of before. There's a sense of freedom. You see something positive."* (Focus group interview 1) |
| **A sense of being needed and available for others** | Finding meaning in late life | Feeling needed | The sense of being needed by others and providing support contributes significantly to overall well-being. | *"No, then you feel unnecessary, and you start to wonder, how will I be needed then? ...I have my youngest grandchild; she is nine now. And then suddenly, she doesn’t want to be picked up by grandma anymore."* (Focus group interview 5) |
|  |  | Meaningful activities | While maintaining engagement in meaningful activities is important, fostering curiosity and exploring new interests and hobbies is equally vital for sustained well-being. | *"You can also engage in hobbies that we didn’t have time for earlier in our working lives. For example, I have started exploring jazz music, so I have a fantastic sound system. I sit for two hours every other day, or almost every day, listening to classic jazz music. It’s quite enjoyable."* (Focus group interview 3) |
|  |  | Social interaction | Engagement in social activities and the maintenance of social networks are essential components of overall well-being. | *"Yes. But then, of course, one needs this network, the social aspect. And it is somewhat up to both oneself and... There is evidently a wide range of options available, both here and there. So yes, it's important to ensure you have a social network before you become too unwell.”* (Focus group interview 2) |
|  |  | Loneliness and companionship | The ability to find satisfaction in solitude, as well as to cultivate meaningful companionship with a partner, is important for emotional well-being. | *"But now life actually feels quite good. I’ve even got a boyfriend."* (Focus group interview 2) |
|  |  | Children and grandchildren | Relationships with one’s children and grandchildren are significant, with shared activities often perceived as particularly meaningful. | *"I often think about what would happen if something were to happen to my children and grandchildren. I wouldn’t be able to continue living as I do because I am so in love with and committed to my family that I would put all my energy into that person. Yes, I can say that. I think about it often. I am grateful if everyone is healthy and employed and so on. I have helped my family tremendously; that has always been important to me."* (Focus group interview 4) |
|  | Others’ perception of aging | Others’ view of old age | Age-related discrimination in societal contexts negatively impacts how aging is perceived and the attitudes held toward older adults. | *”Yes, it is a general perception of how society views older adults. There is a difference: when you go further south in Europe, people have a more positive view of older adults than we do here in the Nordic countries, particularly in Sweden. So, I mean, there is age discrimination."* (Focus group interview 3) |
|  |  | Others’ view of old age | There is a perception of low societal acceptance for activities or processes that may require more time in older age. | *"However, I do believe that sometimes it is important to simply allow an older person to take the time they need, rather than rushing them. It takes time to tap a card and enter a PIN, and instead of standing there sighing impatiently... Of course, I am still fairly quick when making a payment myself, but the point is: let it take time. It also takes time to properly adjust a walker. So, instead of rushing, offer assistance."* (Focus group interview 1) |
|  |  | Societal threats | Experiences of insecurity related to ageist behaviors, such as scams targeting older adults, can adversely affect well-being. | *“Of course, you must be somewhat prepared for this. When someone calls you, you must be very careful.”* (Focus group interview 7) |
|  | Challenges in the welfare system | Healthcare | There is a deterioration in healthcare accessibility, alongside inadequate continuity of care within the healthcare system. | *“…you must go online and book an appointment, and they’ll get back to you. And sure, eventually, you learn how to do it. But at first, I felt, ‘No, I don’t want that, I want to go there and book the appointment’.”* (Focus group interview 3) |
|  |  | Social services | The availability of care homes is insufficient, and concerns remain regarding the quality of care provided. | *“What you're describing, I believe all of us here are thinking about this, how things are in eldercare. And we will most likely all become dependent on it at some point. I think this is something each of us can feel a certain concern about—what will it look like when we are alone and aging.”* (Focus group interview 2) |
